# Characterization of a next-generation piezo bimorph X-ray mirror for synchrotron beamlines

**DOI:** 10.1107/S1600577514020025

**Published:** 2015-01-01

**Authors:** Simon G. Alcock, Ioana Nistea, John P. Sutter, Kawal Sawhney, Jean-Jacques Fermé, Christophe Thellièr, Luca Peverini

**Affiliations:** a Diamond Light Source, Harwell Science and Innovation Campus, Didcot, Oxfordshire OX11 0DE, UK; b Thales-SESO, 305 rue Louis Armand, Pôle d’Activités d’Aix les Milles, Aix-en-Provence, France

**Keywords:** piezo bimorph mirror, synchrotron X-ray optics, active optics, Diamond-NOM, junction effect

## Abstract

Piezo bimorph mirrors are versatile active optics used on many synchrotron beamlines. However, many bimorphs suffer from the ‘junction effect’: a periodic deformation of the optical surface which causes major aberrations to the reflected X-ray beam. This effect is linked to the construction of such mirrors, where piezo ceramics are glued directly below the thin optical substrate. In order to address this problem, a next-generation bimorph with piezos bonded to the side faces of a monolithic substrate was developed at Thales-SESO and optimized at Diamond Light Source. Using metrology feedback from the Diamond-NOM, the optical slope error was reduced to ∼0.5 µrad r.m.s. for a range of ellipses. To maximize usability, a novel holder was built to accommodate the substrate in any orientation. When replacing a first-generation bimorph on a synchrotron beamline, the new mirror significantly improved the size and shape of the reflected X-ray beam. Most importantly, there was no evidence of the junction effect even after eight months of continuous beamline usage. It is hoped that this new design will reinvigorate the use of active bimorph optics at synchrotron and free-electron laser facilities to manipulate and correct X-ray wavefronts.

## Abstract 2

A next-generation bimorph mirror with piezos bonded to the side faces of a monolithic substrate was created. When replacing a first-generation bimorph mirror suffering from the junction effect, the new type of mirror significantly improved the size and shape of the reflected synchrotron X-ray beam. No evidence of the junction effect was observed even after eight months of continuous beamline usage.

## 1. Introduction   

Piezo bimorph mirrors have been used successfully on synchrotron beamlines since the mid-1990s to focus or collimate X-ray beams (Susini *et al.*, 1995 ▶, 1996 ▶; Signorato *et al.*, 1998 ▶). As with mechanically bent mirrors, employing one or two actuators, the optical surface of a bimorph mirror can be bent into a range of ellipses to provide variable size or focal distance of the reflected X-ray beam. Additionally, the high density of piezo actuators (typically 8 to 32) on a synchrotron bimorph mirror also enables low- and mid-spatial frequency errors (period > 20 mm) to be corrected, including polishing defects; gravitational or mounting deformations; photon-induced thermal bumps; and wavefront aberrations caused by non-ideal upstream optics or the X-ray source.

Sixteen bimorph mirrors, manufactured by Thales-SESO, are currently in use on beamlines at Diamond Light Source (DLS). Controls and analysis software scripts, developed to interface with the EPICS control system, are routinely used at all DLS bimorph beamlines including: control of voltages; automatic correction of figure or slope errors; defocusing the X-ray beam to a specified size; automated bending to a specified ellipse; and the creation of non-Gaussian X-ray beam profiles including a constant intensity distribution. Ultimately, the goal of such developments is to make it easier and quicker for beamline users to efficiently use bimorph mirrors. A novel research optic with a slope error of 0.15 µrad r.m.s. for a range of ellipses was also created for DLS by combining a super-polishing technique with bimorph technology (Sawhney *et al.*, 2010 ▶).

**Figure 1:**
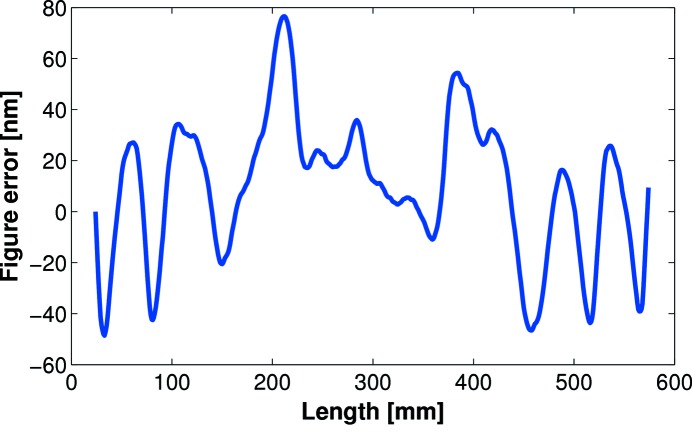
Corrugation of the optical surface on a first-generation bimorph mirror suffering from the junction effect. Extrema occur at the interface between adjacent piezos.

However, many bimorph mirrors at DLS were not fulfilling their potential, so a comprehensive *in situ* (X-ray) and *ex situ* (classical interferometry and slope profilometry) study was initiated by the DLS Optics and Metrology group to investigate and optimize active optics (Sutter *et al.*, 2012 ▶). It was discovered that all first-generation bimorphs at DLS were suffering, with varying degrees of severity, from the ‘junction effect’ (Alcock *et al.*, 2013 ▶), where the optical surface is damaged directly above the interface between adjacent piezo electrodes/ceramic blocks. This leads to a corrugation of the substrate (see Fig. 1 ▶). As the damage shows a high spatial frequency, such defects cannot be corrected using the piezos. The reflected X-ray beam is significantly broadened and distorted by mirrors suffering from the junction effect, leading to a reduction in beamline performance.

**Figure 2:**
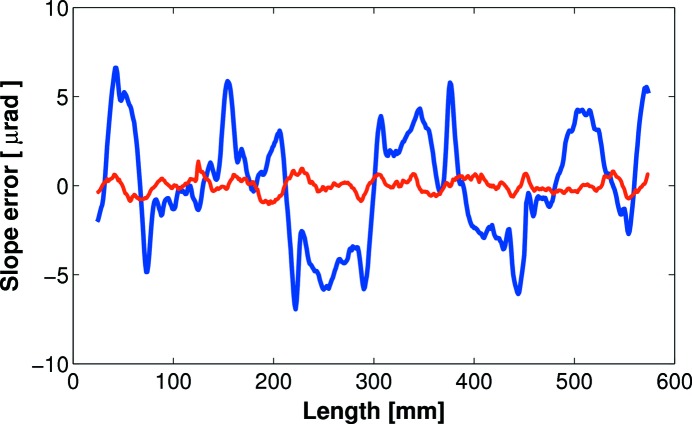
An eightfold improvement to the slope error was achieved by repolishing a first-generation bimorph mirror to remove the junction effect. Before repolishing: 3.1 µrad r.m.s. (thick blue line). After repolishing: 0.4 µrad r.m.s. (thin red line). Comparable results have been achieved with all DLS repolished bimorphs.

To redress these issues, seven of DLS’s bimorph mirrors have been repolished by Thales-SESO. In all cases, repolishing successfully removed the junction effect and greatly reduced the optical slope error, often by a factor greater than 10, to below 0.5 µrad r.m.s. (Fig. 2 ▶). In turn, this led to significant improvements in the size and shape of the reflected X-ray beam profile. Refurbished bimorphs now generate an X-ray focal spot which is close to the theoretical size predicted for the beamline geometry. No degradation in performance has been observed in any of the repolished bimorph mirrors over >18 months of operation. This gives credence to the hypothesis that the junction effect is created soon after production, as the glue, which bonds the piezo ceramics to the substrates, cures. Repolishing solves the problem for existing bimorph mirrors, but for new mirrors a more efficient solution is needed in order to prevent the junction effect from developing and thus avoid this time-consuming and expensive procedure.

## 2. Next-generation bimorph mirrors   

**Figure 3:**
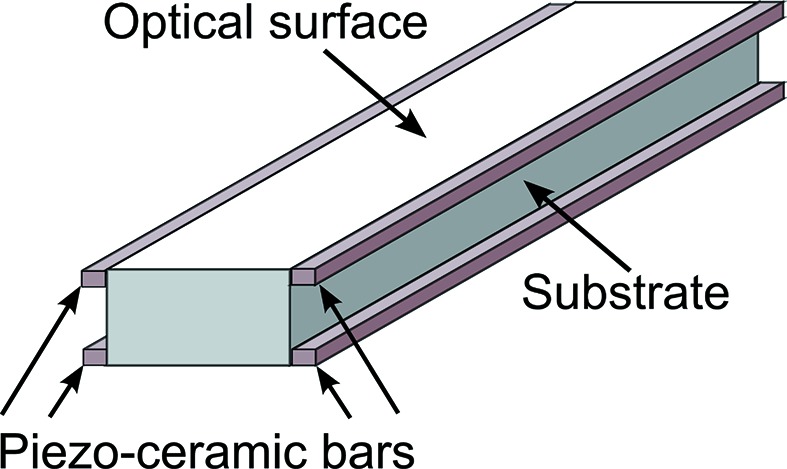
Schematic of the next-generation bimorph mirror from Thales-SESO, showing piezo ceramics glued to the side faces of the monolithic silicon substrate.

In response to feedback from DLS and other synchrotron facilities, a next-generation bimorph mirror with piezo ceramics glued to the side faces of the monolithic substrate (Fig. 3 ▶) was designed by Thales-SESO.

Metallic coatings are applied along the length of each piezo ceramic; non-conductive gaps in the metal coatings create discrete electrodes. When the same voltage is applied to the equivalent electrode on each of the four piezo ceramics, the ceramics locally contract or expand, which imparts mechanical tension or compression to the optical substrate. Since the junction between piezos is no longer directly below the optical surface, it is anticipated that such optics will not suffer from the junction effect. The patented new design (Carré, 2011 ▶) is substantially simpler than the first-generation composite structure which involved gluing numerous piezo ceramic blocks and electrical isolators between optical substrates and spacers (Carré, 2009 ▶). The procedure for constructing a next-generation bimorph also makes it inherently easier to pre-polish monolithic silicon or fused silica substrates using ‘super-polishing’ techniques before piezo ceramics are glued.

Ion beam figuring (IBF) is an attractive technology for creating high-quality optics, particularly non-planar surfaces, for a variety of scientific applications employing a wide range of wavelengths including X-rays (Schindler *et al.*, 2004 ▶; Fruit *et al.*, 1999 ▶). The ion beam is rastered along the mirror with variable speed to preferentially remove material in a profile pre-determined by interferometry or profilometry. Real-time *in situ* X-ray measurement and IBF correction has also been demonstrated (Ziegler *et al.*, 2007 ▶). IBF is typically performed as the final step of fabricating high-quality mirrors (figure errors < 5 nm peak-to-valley), but it can also be used to remove several micrometres of material without degrading micro-roughness (Peverini *et al.*, 2010 ▶). Various types of substrates can be polished by IBF, including Si, SiC (Ghigo *et al.*, 2007 ▶), glasses and ceramics. Some of these materials are difficult to polish using traditional methods. Over the past few years, several optic manufacturers, including Thales-SESO using a scaled-up version of the apparatus described by Peverini *et al.* (2010 ▶), have developed IBF techniques and hardware to profile X-ray mirrors up to 1.5 m in length. The previous design of synchrotron bimorph mirrors was not conducive to ion beam figuring, since the glued joints were susceptible to damage as the substrate’s temperature increased during ion bombardment.

Locating the piezo ceramics away from the direct line of the X-ray beam is also advantageous to minimize radiation damage induced by ultra-intense free-electron laser sources (Yang *et al.*, 2012 ▶). Seemingly, the only disadvantage of next-generation bimorphs is that they are likely to be less responsive to an applied voltage due to the indirect nature of bending, thus reducing the range of curvature. However, as a consequence, smaller-amplitude corrections can be applied for a given voltage, making them ideally suited for X-ray wavefront correction. Bending limitations can be overcome by using a thinner substrate, although sagittal twisting is likely to become a problem for very thin substrates. Alternately, recent advances in manufacturing technologies now enable several profiles, each with a different tangential curvature or figure, to be polished into a single substrate. Rather than applying a large voltage to bend the mirror, the user simply moves the mirror laterally to illuminate an appropriately curved region, and then makes small localized figure error corrections using the piezos.

**Figure 4:**
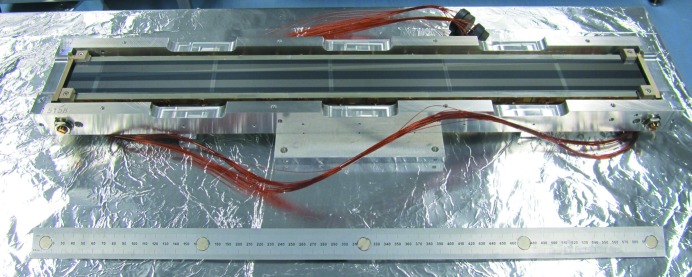
DLS’s next-generation bimorph mirror mounted in a face-upwards geometry.

A next-generation bimorph mirror with 16 electrodes was commissioned by DLS (Fig. 4 ▶). The 640 mm-long mirror is designed to be used on a range of beamlines, and has a versatile mounting scheme enabling it to be oriented facing up, down or sideways for vertical or horizontal focusing. In addition to an uncoated silicon region, rhodium and platinum stripes provide enhanced reflectivity at higher X-ray energies.

## 3. Experimental   

Prior to beamline installation, all synchrotron optics are characterized and optimized in the DLS Metrology cleanroom laboratory using a suite of instruments, including the Diamond-NOM: a non-contact autocollimator-based slope-measuring profiler (Alcock *et al.*, 2010 ▶). The next-generation bimorph mirror was mounted and aligned in a face-upward geometry on the Diamond-NOM, and automated scans were synchronized with application of piezo voltages using EPICS and Python scripts.

### 3.1. Bending range   

**Figure 5:**
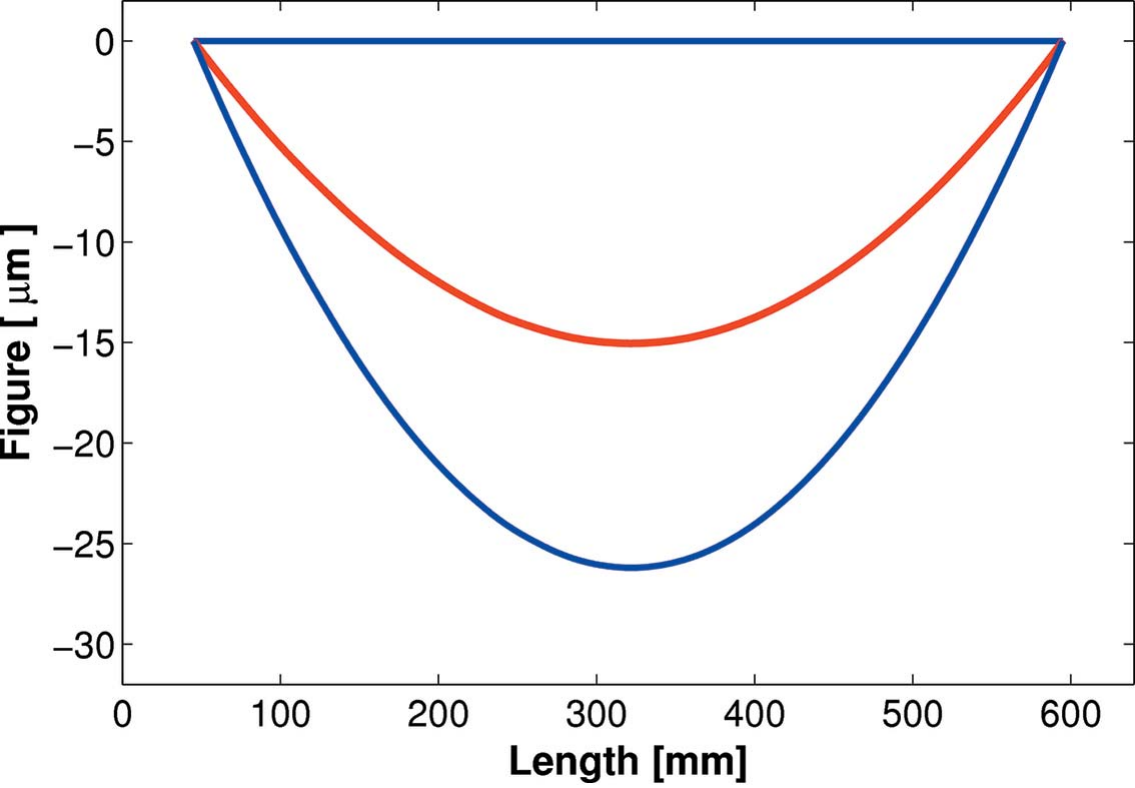
The next-generation bimorph mirror has a dynamic range of curvature from flat (+1300 V) to a concave radius of 1.43 km (−1000 V), making it suitable for a variety of beamline geometries.

To ensure that the mirror was reliably constrained, the bimorph substrate was settled into its holder mechanics by applying several cycles of maximum and minimum voltages to all piezos synchronously. Fig. 5 ▶ shows that the mirror could be made flat by applying +1300 V, or bent to its maximum concave radius of curvature of 1.43 km at −1000 V. Radius of curvature is inversely proportional to applied voltage.

**Figure 6:**
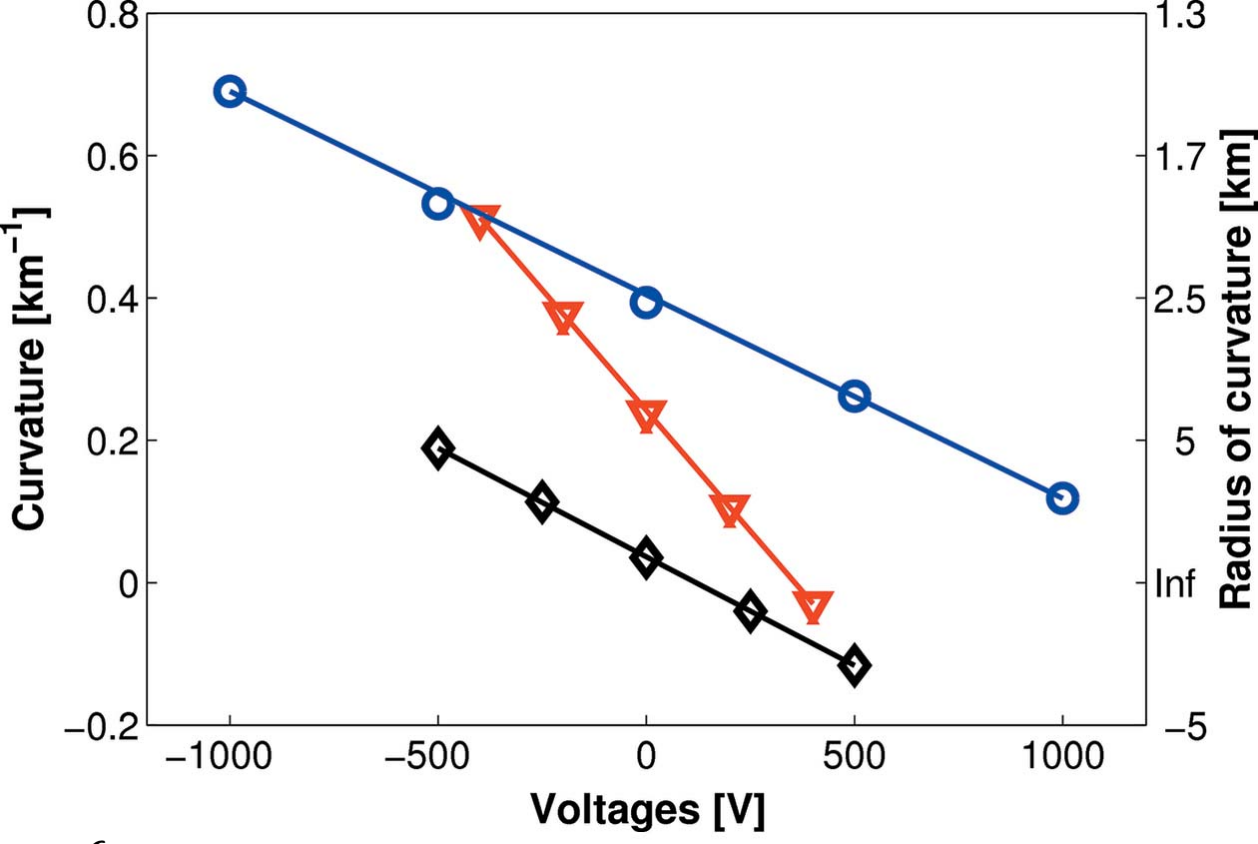
Inverse radius of curvature as a function of applied voltage. A steeper gradient shows that a mirror bends more readily in response to an applied voltage. The next-generation bimorph (blue circles) has a very similar bending rate to a thicker first-generation bimorph (black diamonds), but is less responsive than a first-generation bimorph with comparable thickness (red triangles).

As anticipated, the next-generation bimorph mirror bends less than a classical bimorph of comparable length and thickness (Fig. 6 ▶). However, the rate of bending of the new mirror is very similar to a thicker old-type bimorph, showing that next-generation bimorphs can have comparable bending ranges if the substrate is made marginally thinner.

### 3.2. Stability of bending   

**Figure 7:**
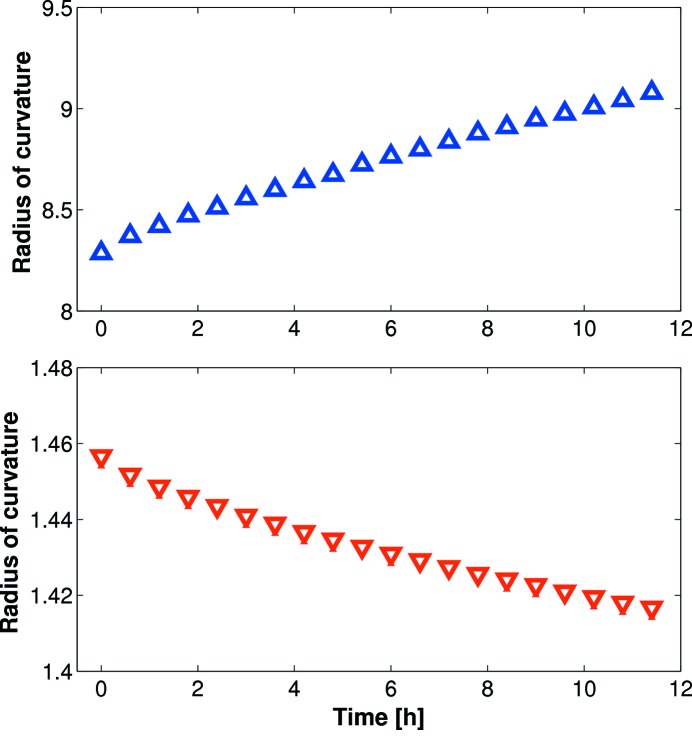
With the mirror holder configured for vertical focusing, over 12 h periods the radius of curvature drifted by ∼10% (upper plot) and ∼3% (lower plot) for maximum positive and negative voltages, respectively. Remounted in a horizontal geometry, with fewer mechanical constraints, the curvature drift was only 0.4%. This gives credence to the hypothesis that the large drifts were caused by non-optimal clamping of the substrate.

Synchrotron X-ray experiments can last for several days, so it is imperative that optics do not change the focal spot size or centroid position during this period. To investigate stability of curvature, the mirror operating in an upward-facing vertical-focusing geometry was bent either to its flattest (+1000 V) or most concave (−1000 V) whilst continually monitoring the radius of curvature using the Diamond-NOM. Unfortunately, even after voltage cycling, over 12 h periods the radius of curvature drifted from 8.291 km to 9.085 km (∼10%) at 1000 V, and from 1.457 km to 1.417 km (∼3%) at −1000 V (Fig. 7 ▶). In both cases the direction of curvature drift was in the direction of bending applied by the piezos: the mirror gradually became flatter for positive voltages and more concave for negative voltages. These drifts can likely be attributed to friction in the kinematic mounts and gravity compensator clamps constraining the substrate. When the mirror was converted to a horizontally focusing geometry, with a reduced number of clamping points, the curvature drift was significantly improved (only ∼0.4% change over a 12 h period). A new design for the holder and kinematic mounts is under consideration.

### 3.3. Piezo response functions   

**Figure 8:**
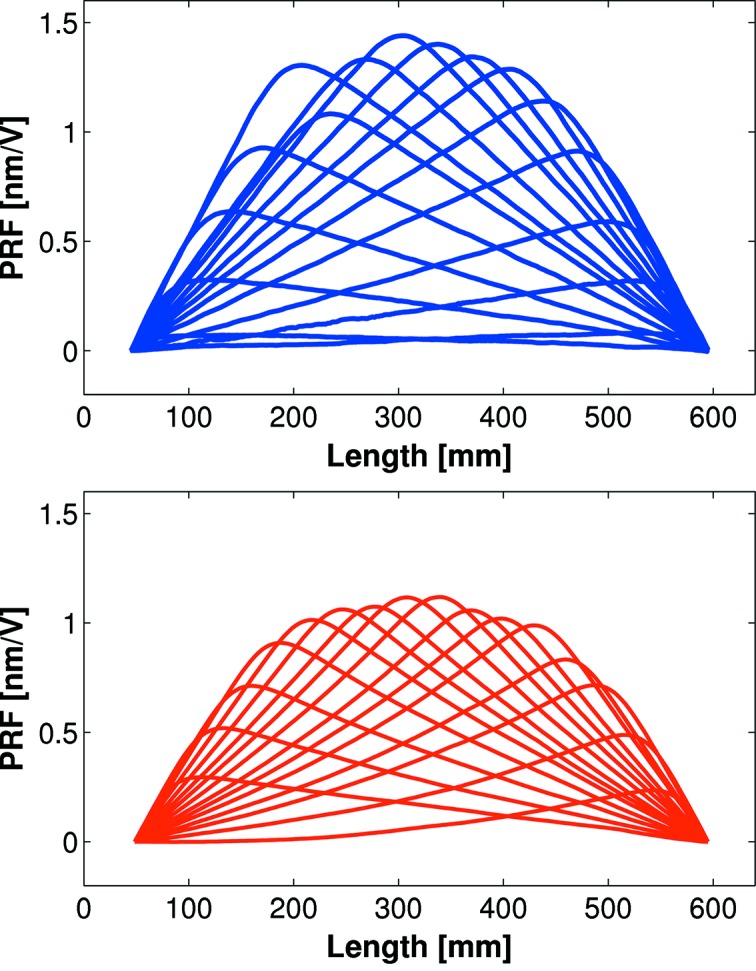
Piezo response functions (PRF) showing how each piezo responds to an applied voltage, as measured using *ex situ* (Diamond-NOM, upper panel) and *in situ* (X-ray, lower panel) methods. Small differences in amplitude can likely be attributed to the different orientation of the mirror in each test.

To characterize how each piezo influences the mirror’s optical surface, a series of piezo response functions (PRFs) were collected by sequentially applying a fixed voltage to each piezo whilst recording how the surface changed using the Diamond-NOM. Previous studies (Alcock *et al.*, 2013 ▶) highlighted that, after applying voltages, sufficient time (typically 20 min, although longer is preferable) needs to be allowed for the surface to stabilize on the nanometre-level before recording each PRF. Fig. 8 ▶ shows that the surface at the centre of the mirror deforms by ∼1.5 nm per applied volt. Since the high-voltage power supply can generate stable voltages with a resolution of 0.1 V, sub-nanometre corrections can easily and reliably be applied to the optical surface. As a comparison, the lower panel of Fig. 8 ▶ shows the same PRFs as measured by *in situ* X-ray methods. The two methods compare very well, with minor discrepancies attributed to: the different orientation of the mirror; operation in air or under ultra-high vacuum; and small uncertainties in scaling factors on the beamline, such as the X-ray angle of incidence.

### 3.4. Minimization of slope/figure error   

**Figure 9:**
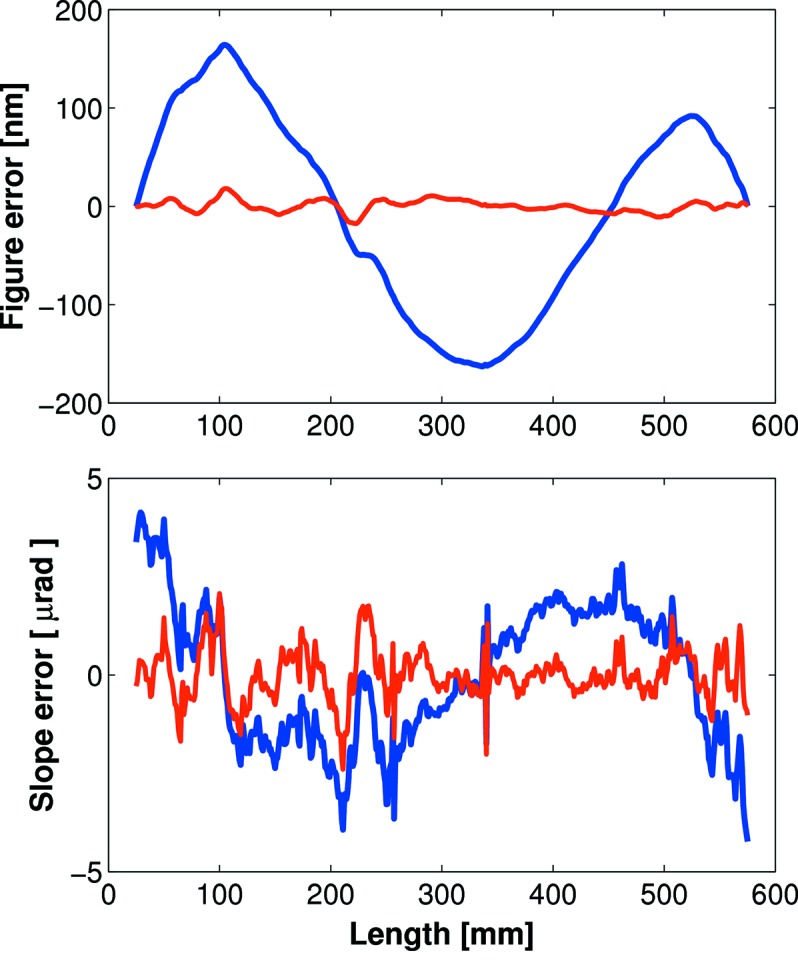
Figure-error (upper panel) and slope-error (lower panel) improvements using a single iteration of the Inverse Matrix algorithm and metrology feedback from the Diamond-NOM. A slope error of ∼0.5 µrad r.m.s. was achieved for a range of ellipses.

Several methods are available to correct optical errors on bimorph mirrors (Huang, 2011 ▶), but perhaps the simplest and most robust is the Inverse Matrix method (Signorato, 1998 ▶; Signorato *et al.*, 1998 ▶). Using this algorithm the slope or figure error to be corrected is decomposed into a linear combination of PRFs; scaling factors correspond to the voltage to be applied to individual piezos. The only constraints are the maximum permissible voltage for all electrodes (typically ±1500 V), and the maximum voltage difference between adjacent piezos (<500 V). In a single iteration of the Inverse Matrix method, the slope error was reduced from ∼2 µrad to ∼0.5 µrad r.m.s. using Diamond-NOM data (Fig. 9 ▶). The corresponding figure error improvement was 106 nm to 4 nm r.m.s. The remaining surface defects are dominated by mid-spatial frequency errors from the polishing process. Such errors have wavelengths shorter than the spacing of the piezos, and hence cannot be further corrected using additional iterations of the Inverse Matrix method.

## 4. Synchrotron X-ray testing   

**Figure 10:**
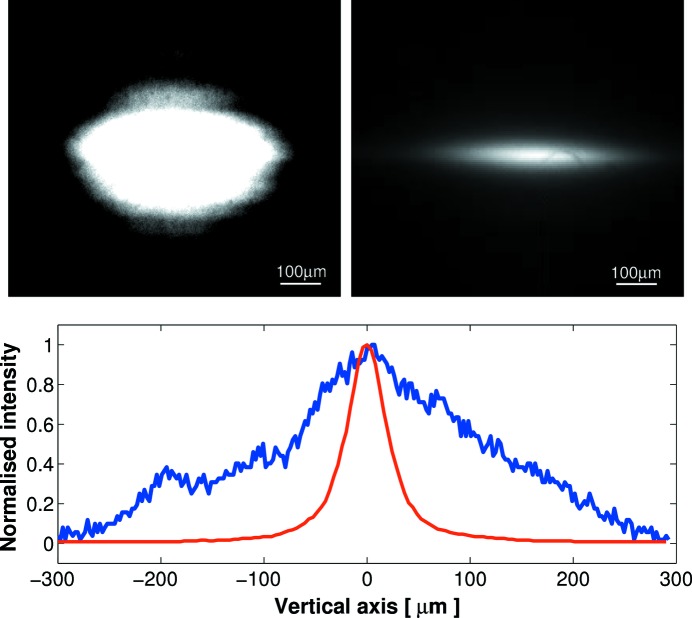
When installed on the I22 beamline in a vertical focusing geometry, the next-generation bimorph mirror produced a much smaller and better defined reflected X-ray beam (upper right-hand image) compared with using the first-generation bimorph mirror suffering from the junction effect (upper left-hand image). The lower chart shows the normalized intensity cross-sectional profile through both X-ray beams in the vertical direction.

After completion of *ex situ* testing using the Diamond-NOM, the next-generation bimorph mirror was reconfigured in its holder and installed in a face-downwards vertical-focusing geometry on the Small Angle Scattering and Diffraction beamline (I22) at DLS. Fig. 10 ▶ shows the X-ray beam profile using I22’s old bimorph mirror (suffering from the junction effect) compared with using the next-generation bimorph. A significant improvement to the size and shape of the X-ray focal spot was achieved using the next-generation bimorph mirror: the vertical X-ray beam size was reduced from 183 µm to 44 µm FWHM, which is in good agreement with the value predicted by the beamline geometry.

**Figure 11:**
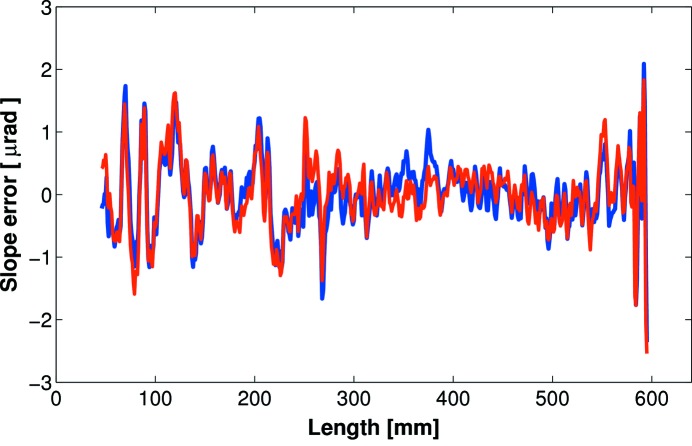
Diamond-NOM scans of the next-generation bimorph mirror before (blue) and after (red) eight months of beamline usage, showing that the junction effect has not appeared.

Throughout eight months of constant beamline usage, the X-ray performance remained consistently good. After removal from the beamline, the mirror was re-scanned using the Diamond-NOM. As anticipated, there was no change in the surface topography and no evidence of the junction effect (Fig. 11 ▶).

## 5. Conclusions   

A metrology study was performed to characterize a next-generation bimorph mirror procured from Thales-SESO. The new design makes it significantly easier to polish a monolithic substrate using super-polishing techniques prior to gluing piezo ceramics to the side faces of the substrate. Sixteen piezos along the 640 mm-long optic enable the surface to be locally controlled with sub-nanometre precision, and bent from flat to 1.4 km concave. Using feedback from the Diamond-NOM, the slope error was reduced to ∼0.5 µrad r.m.s. for a range of ellipses and cylinders. As anticipated, the next-generation bimorph mirror bends less than a first-generation mirror of comparable length and thickness. Interestingly, the rate of bending of the new mirror was almost identical to a thicker first-generation bimorph mirror, showing that next-generation bimorphs can have extended bending ranges if the substrate is sufficiently thin. A novel holder was fabricated to enable the mirror to operate in vertical or horizontal focusing modes. However, friction within the kinematic mounts and clamps meant that the curvature of the mirror drifted by up to 10% in extreme cases. Once installed on a beamline, the mirror generated a significantly smaller and better defined X-ray focal spot compared with using an old bimorph mirror suffering from the junction effect. The most significant aspect of this investigation was to confirm the absence of the junction effect for the next-generation bimorph, even after eight months of constant beamline usage. These encouraging results may help to rebuild the synchrotron community’s trust in the use of piezo bimorph mirrors to provide high-quality X-ray beams of variable focal size and shape.
